# Peptidomic Profiling Analysis of Endogenous Peptides in Buffalo Milk During Lactation Stages

**DOI:** 10.3390/foods15101728

**Published:** 2026-05-14

**Authors:** Yue Zhang, Xingchen Huang, Rongchun Huang, Pingbai Liu, Jiazheng Zhu, Yuan Yang, Gan Liang, Meiting Chen, Mengyuan Zhou, Guangsheng Qin, Qiang Fu

**Affiliations:** 1 College of Animal Science and Technology, Guangxi University, Nanning 530004, China; zhangyue200002@163.com (Y.Z.); xchuang@st.gxu.edu.cn (X.H.); 18386484298@163.com (P.L.); ggbond0330@163.com (J.Z.); 18211816773@163.com (Y.Y.); 2 Buffalo Research Institute, Chinese Academy of Agricultural Sciences, Nanning 530001, China; 18977545979@163.com (R.H.); 18776452585@163.com (G.L.); 2112159161@stu.fosu.edu.cn (M.C.); 18900296501@163.com (M.Z.)

**Keywords:** buffalo milk, endogenous peptides, peptidomics, colostrum, anti-inflammatory peptides

## Abstract

Buffalo milk is a rich source of various nutritional components and bioactive peptides, offering significant health benefits. Endogenous peptides, which occur naturally in milk, represent a valuable source of bioactive peptides with potential nutraceutical applications. However, research on endogenous peptides in buffalo milk remains limited. This study employed a quantitative peptidomic approach to characterize endogenous peptides across different lactation stages. A total of 2099, 2946, and 4418 peptides were identified in colostrum, transitional milk, and mature milk, respectively. The majority of these peptides were derived from β-casein, followed by α_S1_-casein, κ-casein, and other proteins. Notably, variations in precursor proteins contributing to peptide production were observed throughout lactation. Phosphorylation levels of endogenous peptides were highest in mature milk, with serine residues predominating. Enzymatic cleavage analysis identified cathepsin D as the key enzyme involved in endogenous peptide production, while proline endopeptidase and plasmin exhibited stage-specific activities. Bioinformatics analysis revealed differentially expressed precursor proteins linked to complement cascades and NF-κB signaling, emphasizing the immune protective role of colostrum. Furthermore, 54 potentially bioactive peptides with favorable water solubility were identified in colostrum, of which 17 were predicted to possess anti-inflammatory properties. These findings contribute to a deeper understanding of the molecular basis of buffalo milk’s functional properties, highlighting its potential as a source of bioactive peptides for both nutritional and pharmaceutical applications.

## 1. Introduction

Buffalo (*Bubalus bubalis*) milk exhibits a more favorable nutritional profile than cow milk, characterized by higher energy content, lower cholesterol, and elevated concentrations of proteins and calcium [1,2]. The total dry matter (157~172 g/L) and energy content (4244~4479 kJ/L) in buffalo milk are higher than those of cow milk (118~130 g/L; 2709~2843 kJ/L) [3]. Specifically, the lipid profile of buffalo milk is similar to that of cow milk, but the fat content in buffalo milk is approximately twice as high as that in cow milk [4]. Buffalo milk contains 4.06 to 4.46% protein content (27~52 g/L), which is higher than that of cow milk (3.2% on average, 29~37 g/L) [1,4]. Additionally, buffalo milk whey contains natural constituents, such as immunoglobulins, lactoferrin, and growth factors, which are considered a prominent source for nutraceutical applications [5,6]. These unique characteristics make buffalo milk an important dietary component and an excellent alternative or supplement for infant nutrition [7,8].

Colostrum is the initial mammary secretion post-parturition, bestowing a complete nutritional profile and bioactive compounds vital for optimum nutrition and promoting the growth, development, and immunological defense of newborns [9]. The composition, structure, and physicochemical properties fluctuate significantly during the transition from colostrum to mature milk. Colostrum contains high concentrations of bioactive compounds, particularly immunoglobulin G (IgG), to provide passive immunity. In addition, colostrum is rich in growth factors, lactoperoxidase, lysozyme, lactoferrin, cytokines, vitamins, and peptides. These components engage in biological processes, including maturation of the gastrointestinal tract, immune response, energy homeostasis, and protection against pathogens [10]. Bioactive components of colostrum possess cross-species bioactivity, making it an attractive choice in the research and development of functional foods and pharmaceuticals with veterinary and human applications [11,12]. Studies on colostrum have been conducted in humans [13,14], cows [15], sheep [16], and buffalo [17]. Our previous findings indicated that buffalo colostrum proteins actively enhance nascent immunity, particularly through interleukin and interferon signaling pathways [17].

Milk proteins are recognized as valuable and abundant sources of specific bioactive peptides, owing to their diverse physiological activities [18]. Bioactive peptides in milk originate from two distinct sources: exogenous peptides and endogenous peptides. Exogenous peptides are typically produced by in vitro enzymatic hydrolysis or microbial fermentation. In contrast, endogenous peptides exist natively in the mammary gland without any artificial processing [19,20]. Previous reports demonstrated that milk contains hundreds of naturally occurring peptides released by plasmin and other milk proteases within the mammary gland. 18% of endogenous peptides in human milk have over 50% homology with known functional peptides, and a large proportion align with known antimicrobial peptides [21,22]. Casein represents the predominant precursor protein for the generation of endogenous peptides [23]. Endogenous peptides in human milk have been confirmed to exert versatile biological activities, including antimicrobial, antioxidant, and immunomodulatory effects [24,25,26]. In mammals, endogenous peptides isolated from camel milk and donkey milk display potential dipeptidyl peptidase IV (DPP-IV) inhibitory, antioxidant, and angiotensin-converting enzyme (ACE) inhibitory activities [27,28]. Similarly, endogenous peptides in bovine milk possess antihypertensive, antimicrobial, mineral-binding, and intestinal regulatory properties [29]. Notably, dynamic changes in endogenous peptides across distinct lactation stages have been documented in bovine milk and donkey milk, and these stage-dependent changes are closely associated with nutritional supply [27,29]. However, the endogenous peptide profiles of buffalo milk remain uncharacterized. Thus, understanding the dynamic changes in endogenous peptides is crucial for uncovering the potential biological significance of buffalo milk.

Peptidomics, which focuses on analyzing peptides in native forms [30], is an emerging field derived from proteomics and enabled by modern separation, analytical, and computational technologies [31]. This study employed a peptidomic approach based on liquid chromatography coupled with tandem mass spectrometry (LC-MS/MS) to comprehensively analyze and compare the endogenous peptides in buffalo colostrum, transitional milk, and mature milk. This approach offers high sensitivity and throughput, facilitating the identification of novel bioactive peptides and providing deeper insights into the functional roles of endogenous peptides [32,33]. These findings significantly enhance our understanding of the peptide composition in buffalo milk and provide a foundation for discovering novel bioactive peptides from buffalo colostrum.

## 2. Material and Methods

### 2.1. Sample Collection

**Figure 1:**
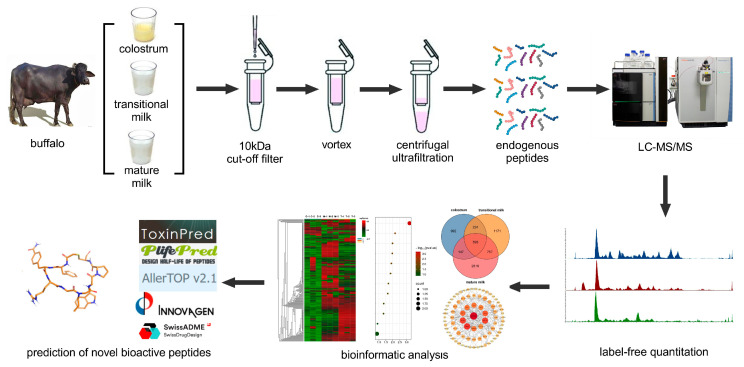
Scheme of analytical workflow for peptidomics and bioinformatics.

Fresh buffalo milk samples were collected from 18 healthy Murrah buffaloes (aged 2–3 years, weighing 300–400 kg) at the Guangxi Buffalo Institute of the Chinese Academy of Agricultural Sciences. Colostrum (BC), transitional milk (BT), and mature milk (BM) samples were obtained on the 1st, 7th, and 30th days postpartum, respectively. The 18 samples were randomly divided into three groups. The experimental workflow is shown in Figure 1.

### 2.2. Peptide Ultrafiltration

Milk samples were centrifuged at 3800 rpm for 20 min at 4 °C to remove the upper fat layer and sediment. The caseins were removed as previously described [17]. Briefly, the supernatant was adjusted to pH 4.6 with 10% acetic acid and mixed with an equal volume of 20% (*w*/*v*) trichloroacetic acid (TCA) for 30 min at 4 °C. The samples were then centrifuged at 14,000× *g* for 30 min at 4 °C to precipitate caseins. The resulting supernatant was collected and filtered using a 10 kDa cutoff ultrafiltration tube (Merck Millipore, Burlington, MA, USA). The ultrafiltration tubes were rinsed twice with milli-Q water. The supernatant was then loaded onto the inner tube and centrifuged at 4000× *g* for 10 min to collect the peptides in the outer tube. The peptides collected from the outer tube were concentrated using a centrifugal vacuum concentrator (Eppendorf, Concentrator plus, Hamburg, Germany) and desalted using ZipTip C18 tips (Cat No. ZTC18S096, Merck Millipore, Burlington, MA, USA) according to the manufacturer’s instructions.

### 2.3. LC-MS/MS Analysis

Peptide identification was performed using nano-liquid chromatography (nano-LC) coupled with an Orbitrap Fusion mass spectrometer (Thermo Fisher Scientific, Waltham, MA, USA), as previously described [34]. Briefly, the extracted peptides were dissolved in 10 μL of solvent A (2% ACN, 0.1% TFA). The peptides were loaded onto a reverse-phase trap column (Acclaim PepMap 100 C18, 3 μm, 100 Å, 75 μm × 2 cm, Thermo Fisher Scientific, Waltham, MA, USA) and transferred to a reversed-phase analytical column (Acclaim PepMap RSLC C18, 2 μm, 100 Å, 50 μm × 15 cm, Thermo Fisher Scientific, Waltham, MA, USA) at a flow rate of 300 nL/min. The peptides were eluted from the column with a 115 min gradient elution of solvent B (98% ACN, 0.1% TFA), 5% to 25% solvent B for 60 min, 25% to 35% solvent B for 40 min, 35% to 55% solvent B for 10 min, 55% to 100% solvent B for 5 min. Eluted peptides were ionized by a Nanospray Flex Ion Source (Thermo Fisher Scientific, Bremen, Germany). The acquired precursor ions ranging from 300~1800 m/z were detected using an orbitrap analyzer at a resolution of 70,000. The top 20 most abundant precursor ions were selected for fragmentation with an isolation window of 2 m/z. Dynamic exclusion was set to 45 s, and ion 445.1200 (*m*/*z*) was used as an internal standard. All MS experiments were repeated thrice to ensure accuracy and reproducibility.

### 2.4. Data Processing and Bioinformatic Analysis

Raw data were analyzed against *Bos taurus* (Uniprot accession No. 000009136, released in April 2024, 51,509 sequences, http://www.uniprot.org/proteomes/UP000009136, accessed on 9 November 2024) using PEAKs Studio software (v12.0, BS Inc., Calgary, AB, Canada). Mass error tolerance was set to 20 ppm for precursor ions and 0.02 Da for product ions. No enzymatic digestion was specified, with variable modifications including acetylation (N-terminal, +42.01 Da), oxidation (M, +15.99 Da), and phosphorylation (STY, +79.97 Da), allowing a maximum of two modifications per peptide. Peptides were filtered at a 1% false discovery rate (FDR), unique peptides ≥ 1, de novo ALC ≥ 50%. Differentially expressed peptides were quantified using a database search algorithm. Gene Ontology (GO) analysis was conducted using KOBAS 3.0 software (http://bioinfo.org/kobas, accessed on 15 November 2024) [35] with a corrected *p*-value < 0.05. Pathway annotations were retrieved from the KEGG database (https://www.kegg.jp/, accessed on 18 November 2024) [36]. Protein–protein interaction networks were constructed using the STRING database (https://string-db.org/, accessed on 23 November 2024) [37] and visualized with Cytoscape 3.2 software. Statistical and visualization analyses were performed using GraphPad Prism 8 software.

### 2.5. Functional Analysis of Endogenous Peptide

To explore the natural formation mechanism and potential bioactivities of the identified endogenous peptides, bioinformatic analyses were performed. Protease prediction was performed using Enzyme Predictor (http://bioware.ucd.ie/~enzpred/Enzpred.php, accessed on 5 December 2024) [38] to identify potential proteases that generate the natural endogenous peptides in vivo cleavage of precursor proteins in the mammary gland. The biological activity potential of identified peptides was assessed using Peptide Ranker (https://peptide.ucd.ie/), with higher scores indicating a greater likelihood of bioactivity [39]. Peptide toxicity and physicochemical properties, including hydrophilicity, hydrophobicity, steric hindrance, charge, isoelectric point, and molecular weight, were predicted using ToxinPred (https://webs.iiitd.edu.in/raghava/toxinpred, accessed on 9 December 2024) [40]. Water solubility was evaluated using Innovagen tools (https://pepcalc.com/, accessed on 13 December 2024). Finally, potential bioactive peptides were compared against the Milk Bioactive Peptide Database (MBPD) [41] and BIOPEP-UWM [42] to identify previously reported bioactive peptides.

## 3. Results

### 3.1. Profiles of Endogenous Peptides in Buffalo Milk

**Figure 2:**
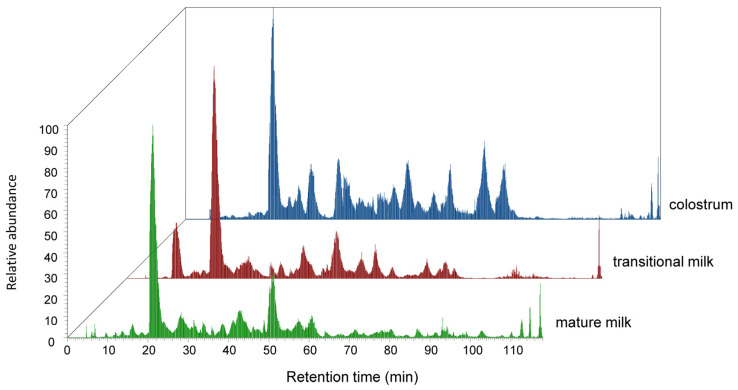
Total ion chromatograms of endogenous peptides in colostrum, transitional milk and mature milk.

A data-dependent acquisition (DDA) mode with LC-MS/MS was employed to investigate endogenous peptides in BC, BT, and BM. Under identical liquid chromatography conditions, the total ion chromatogram (TIC) of all three sample types displayed a substantial number of overlapping peaks (Figure 2). In the BT and BM groups, most peptides were eluted between 20~60 min, whereas the TIC of the BC group showed a distinct profile, with more hydrophobic peptides eluted between 60~90 min. The total ion intensities were 2.79 × 10^9^, 4.25 × 10^9^, and 5.76× 10^9^ in BC, BT, and BM, respectively. Notably, the MS signal intensity in the BM group was approximately twice as high as that in the BC group, suggesting a progressive increase in peptide content during lactation. The raw data of milk endogenous peptidome can be fully accessed from the iProX (IPX0016553001), an official member of the ProteomeXchange consortium [43].

### 3.2. Comparisons of Peptidomic Profiles in Buffalo Milk

**Figure 3:**
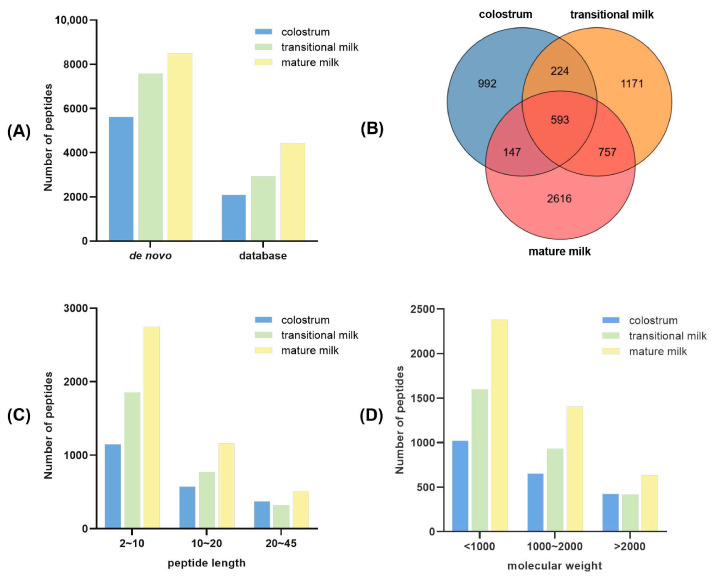
Characterization of endogenous peptides identified by LC-MS/MS. (**A**) Comparison of endogenous peptides identified by database search and de novo algorithms. (**B**) Venn diagram of endogenous peptides of BC, BM and BT. (**C**) Distribution of peptide length. (**D**) Distribution of peptide molecular weight.

Endogenous peptides were identified using two approaches: database search and de novo algorithms. The FDR curve and scatterplot of precursor mass error (Supplementary File S1) validated the high confidence of all identified peptides. In total, 2099, 2946, and 4418 endogenous peptides were identified in the BC, BT, and BM groups, respectively, through database search. As shown in Figure 3A, de novo identification revealed additional peptides not found in the database (Supplementary Table S1). These results indicate that mature milk contains a greater number and abundance of endogenous peptides compared to colostrum. Abundant endogenous peptides were detected in mature milk, which mainly originated from sustained proteolytic hydrolysis of milk proteins by endogenous proteases in the mammary gland, reflecting the physiological transition from immune-dominated secretion in colostrum to homeostatic proteolytic metabolism in mature milk.

A Venn diagram was generated to analyze the differences in peptide expression among the BC, BT, and BM groups. The diagram revealed that 740 peptides were shared between BC and BM, 817 between BC and BT, and 1350 between BT and BM. Only 593 peptides (28.3% of the colostrum peptidome) were common across all three groups. Notably, 992 endogenous peptides were unique to colostrum, while 2616 peptides (59.2% of the mature milk peptidome) were exclusive to mature milk (Figure 3B), indicating that mature milk produces a higher abundance of endogenous peptides.

The physicochemical properties of the peptides, including molecular weight and peptide length, were similar across all three groups. The majority of peptides ranged from 2 to 10 amino acids in length (Figure 3C), with average lengths of 12, 10, and 10 amino acids in BC, BT, and BM, respectively. The molecular weight of endogenous peptides was predominantly below 10 kDa (Figure 3D). The average peptide mass was 1332.1 Da, 1161.9 Da, and 1160.5 Da in BC, BT, and BM, respectively, suggesting that peptides in mature milk have lower molecular weights compared to colostrum.

### 3.3. Modification of Endogenous Peptide

**Figure 4:**
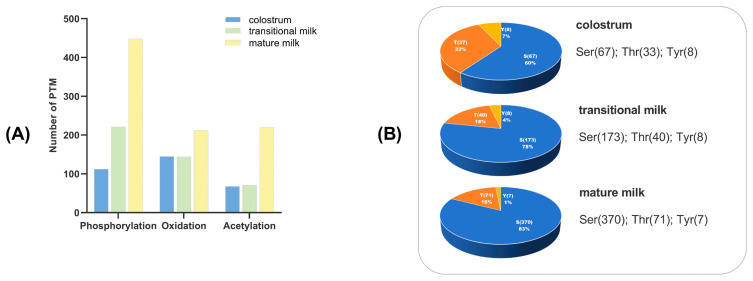
Post-translational modification analysis of endogenous peptides. (**A**) Number of peptide modification sites in buffalo milk. (**B**) Distribution of phosphorylation sites.

Peptide modifications, including acetylation, oxidation, and phosphorylation, were analyzed based on mass shift and phosphate group neutral loss scanning. The number of phosphopeptides in BC was lower than that in BT and BM. In terms of proportion, serine (Ser) was the most phosphorylated amino acid residue, accounting for over 60% of the phosphorylated residues in all groups. Threonine (Thr) phosphorylated amino acid residues represented 33%, 18%, and 16% in BC, BT, and BM, respectively. A total of 99 phosphorylation sites on 47 phosphoproteins were identified in BC, increasing to 181 phosphorylation sites on 54 phosphoproteins in BT and 366 sites on 102 phosphoproteins in BM (Figure 4). These results highlight that phosphorylation levels are highest in mature milk.

### 3.4. Precursor Proteins of Endogenous Peptides

**Figure 5:**
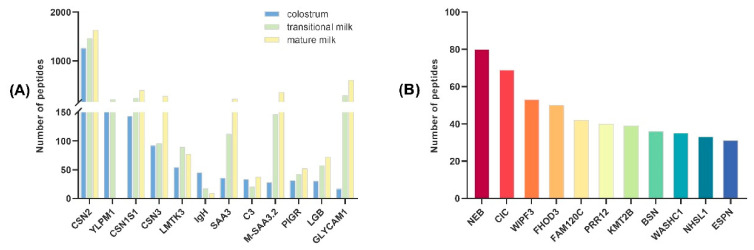
Identification of main precursor proteins of buffalo milk. (**A**) Major precursor proteins for endogenous peptides. (**B**) Unique precursor proteins of endogenous peptides in buffalo colostrum.

A total of 6500 endogenous peptides derived from 358 precursor proteins were identified across the three groups (Supplementary Table S2). As shown in Figure 5A, β-casein (CSN2) was the primary source of endogenous peptides, followed by α_S1_-casein (CSN1S1) and κ-casein (CSN3). CSN2 contributed 555, 649, and 750 endogenous peptides in BC, BT, and BM, respectively. Notably, the contribution of precursor proteins to endogenous peptide production showed considerable variations during lactation. The YLP motif containing 1 (YLPM1) contributed more than 150 endogenous peptides in BC and BT but was absent in BM. Conversely, serum amyloid A (SAA), mammary serum amyloid A3.2 (M-SAA3.2), and glycosylation-dependent cell adhesion molecule 1 (GLYCAM1) contributed significantly more peptides in BT and BM than in BC. The Ig-like heavy chain protein (IgH) produced more than 50 endogenous peptides in BC, but was scarcely found in BT and BM. Particularly, unique precursor proteins in colostrum, such as Nebulin (NEB), Capicua transcriptional repressor (CIC), WAS/WASL interacting protein family member 3 (WIPF3), Formin homology 2 domain containing 3 (FHOD3), Family with sequence similarity 120C (FAM120C), Proline-rich 12 (PRR12), and others, contributed 508 endogenous peptides (Figure 5B). These results demonstrated significant differences in the precursor proteins of endogenous peptides across different lactation stages.

### 3.5. Proteolytic Cleavage Sites of Precursor Proteins

**Figure 6:**
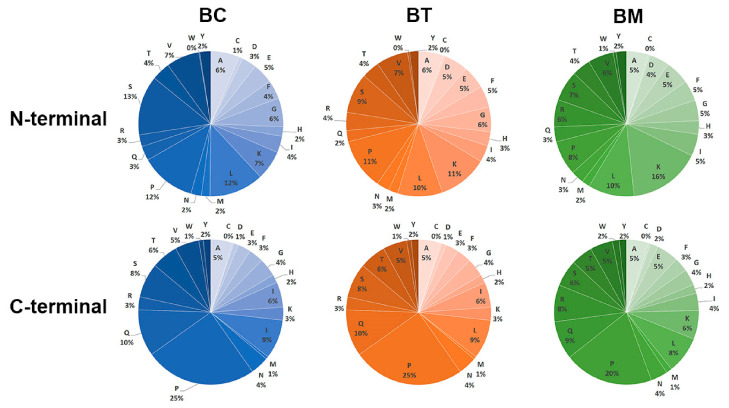
Distribution of amino acid residues of N- and C-terminal cleavage sites.

To investigate the proteolytic cleavage profiles of precursor proteins in buffalo milk, the amino acid positions and distribution at the N- and C-terminals of identified endogenous peptides were analyzed (Supplementary Table S3). As shown in Figure 6, N-terminal residues in the BC group were primarily serine (13%), proline (12%), and leucine (12%), while C-terminal residues were predominantly proline (25%) and glutamine (10%). Similar profiles were observed in the BT and BM groups. In BT, N-terminal residues were mainly proline (11%), lysine (11%), and C-terminal residues were predominantly proline (25%) and glutamine (10%). In BM, N-terminal residues were primarily lysine (16%) and leucine (10%), while C-terminal residues were mostly proline (20%) and glutamine (9%). These results indicate significant differences in N-terminal residues during lactation, while C-terminal residues remain relatively consistent. Additionally, 28 potential enzymes responsible for proteolytic cleavage were predicted using Enzyme Predictor. Cathepsin D and proline endopeptidase were identified as the most likely enzymes in BC and BT, while cathepsin D and plasmin were predominant in BM. These results suggest that Cathepsin D contributes to the generation of endogenous peptides throughout lactation, with proline endopeptidase and plasmin exerting their functions at distinct periods of lactation.

### 3.6. Quantitative Analysis of Precursor Proteins

**Figure 7:**
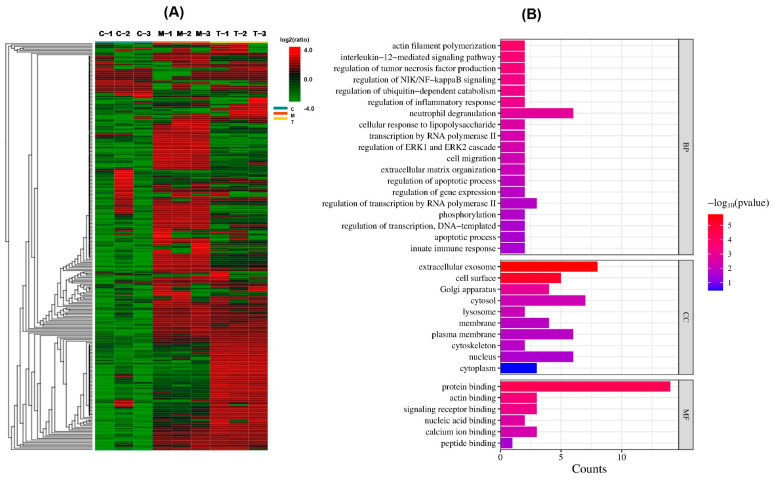
Heatmap and GO annotation of the DEPPs. (**A**) Heatmap of precursor protein expression. (**B**) GO classification of DEPPs.

To analyze the differentially expressed precursor proteins (DEPPs) among the BC, BT, and BM groups, a label-free peptidomic analysis based on peak area from LC-MS/MS was performed. A total of 2016 peptides, belonging to 219 precursor proteins with quantitative information, were successfully identified across the three groups. The complete peptide lists, including amino acid sequences, precursor protein names, and peak areas, are presented in Supplementary Table S4. As shown in Figure 7A, a heatmap of precursor protein expression levels demonstrated high accuracy and reproducibility across biological replicates. Compared to the BM group, a total of 1015 differentially expressed endogenous peptides (fold change ≥ 5 or ≤0.2) corresponding to 116 precursor proteins were characterized in the BC group, including 283 upregulated peptides and 732 downregulated peptides, indicating a general decline trend in endogenous peptide expression in buffalo colostrum.

### 3.7. Gene Ontology Analysis

To investigate the potential physiological functions of the DEPPs, enriched GO terms were classified into three categories: Cellular Component (CC), Biological Process (BP), and Molecular Function (MF) (Supplementary Table S5). As shown in Figure 7B, the primary annotations for each category are illustrated. In terms of CC, GO classification revealed that DEPPs were primarily located in extracellular exosomes, cell surface, Golgi apparatus, cytosol, membrane, and cytoskeleton of cellular components, suggesting that precursor proteins are mainly derived from transmembrane transport. Regarding BP, numerous DEPPs were primarily involved in actin filament polymerization, interleukin-12-mediated signaling, cellular response to lipopolysaccharide, regulation of the ERK1 and ERK2 cascade, cell migration, phosphorylation, apoptotic process, and regulation of inflammatory response (Figure 7B).

### 3.8. KEGG Enrichment and PPI Network Analysis

**Figure 8:**
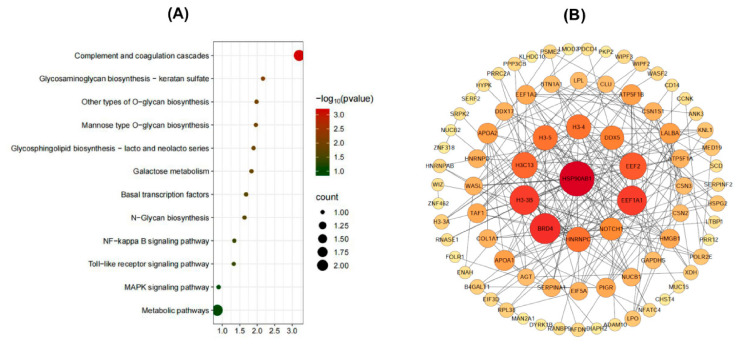
KEGG pathway and PPI network analysis of DEPPs. (**A**) KEGG pathway enrichment of DEPPs. (**B**) PPI networks of DEPPs.

KEGG enrichment analysis revealed that 116 DEPPs were associated with 15 metabolic pathways (Supplementary Table S6). The top 12 pathways are presented in Figure 8A. The DEPPs were primarily involved in the complement and coagulation cascades, glycosaminoglycan biosynthesis, O-glycan biosynthesis, N-glycan biosynthesis, glycosphingolipid biosynthesis, and galactose metabolism, among others. Several key signaling pathways, including NF-κB signaling, Toll-like receptor signaling, and MAPK signaling, were significantly enriched, suggesting a central role of DEPPs in immune protection. Particularly, the complement and coagulation cascades, associated with the innate immune system, highlight how buffalo colostrum enhances immune defense to provide rapid protection against infections.

Additionally, protein–protein interaction (PPI) networks were constructed to identify hub precursor proteins using Cytoscape 3.2 software. The PPI network contained 82 nodes and 454 edges (Figure 8B). The top crucial proteins maintaining the stability of the PPI network included HSP90AB1, BRD4, H3-3B, EEFEA1, EEF2, NOTCH1, HNRNPC, H3C13, H3-4, H3-5, and DDX5 (Figure 8B). The node with the most interactions was HSP90AB1, interacting with 22 proteins, followed by BRD4 with 18 interactions, and H3-3B and EEFEA1, each with 17 interactions.

### 3.9. In Silico Analysis of Potential Bioactive Peptides in Buffalo Colostrum

**Figure 9:**
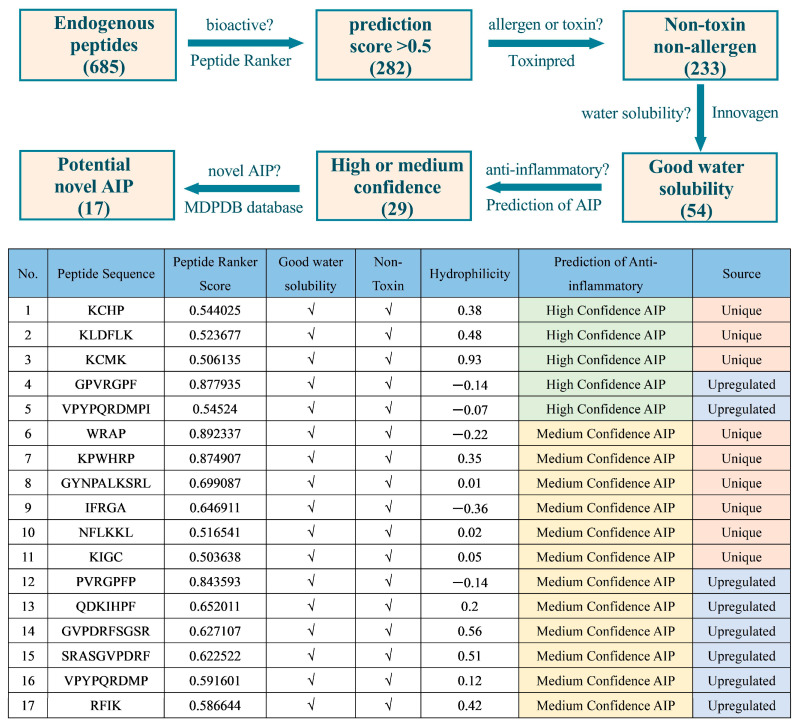
In silico strategy for the screening of potential anti-inflammatory peptides.

To further identify potential novel bioactive peptides in buffalo colostrum, an in silico screening strategy was employed on the identified peptides (Figure 9). A total of 685 endogenous peptides (580 unique peptides and 105 upregulated peptides) from buffalo colostrum were evaluated using the Peptide Ranker tool to assess their activity scores. Of these, 282 peptides received scores greater than 0.5, indicating a higher likelihood of biological activity. Additionally, potential allergenicity and toxicity were predicted to exclude potentially harmful peptides. The results showed that 233 peptides were predicted to be non-allergenic and non-toxic. Water solubility, a key factor influencing peptide bioavailability, was also assessed. Among the 233 non-toxic and non-allergenic peptides, 54 exhibited favorable water solubility (Supplementary Table S7). Furthermore, 17 peptides were predicted to have potential anti-inflammatory bioactivities based on the anti-inflammatory database.

## 4. Discussion

Endogenous peptides, typically present at low concentrations in milk, pose significant challenges for identification and analysis. With the rapid development of LC-MS/MS, peptidomics has emerged as the gold standard for the identification of peptides and their precursor proteins [23,44]. This study presents a comprehensive peptidomic analysis comparing endogenous peptides across three lactation stages in buffalo milk. A key finding is the elevated abundance of endogenous peptides identified through ultrafiltration and high-resolution LC-MS/MS techniques. Enhanced peptide coverage is attributed to the utilization of a DDA mode with optimized LC-MS/MS parameters, as well as the combined use of both database search and de novo identification approaches. A significant number of endogenous peptides were identified and quantified, revealing dynamic changes during buffalo lactation. The progressive increase in peptide abundance from colostrum to mature milk corroborates prior findings in bovine milk [45], suggesting a conserved mechanism of peptide generation during lactation. However, this study uncovers a unique profile in buffalo colostrum, characterized by a higher proportion of hydrophobic peptides, which elute at later retention times. These hydrophobic peptides may enhance stability and bioavailability, potentially contributing to the superior nutritional and immunological properties of buffalo colostrum. This is, to our knowledge, the first study to detail the endogenous peptide profile of buffalo milk. The results not only provide a molecular basis for functional peptide research but also highlight the potential applications of bioactive peptides in buffalo milk.

Unlike peptides derived from protein digestion, endogenous peptides may exert biological functions independently of gastrointestinal processes [46,47]. Previous studies have demonstrated that short peptides can enter the cellular nucleus and interact with nucleosomes, histones, and gene promoters [48,49]. In this study, short-chain endogenous peptides (fewer than 10 amino acids) represent 54.8%, 62.9%, and 62.2% of the peptides in colostrum, transitional milk, and mature milk, respectively. From a nutritional standpoint, short peptide sequences are less likely to undergo in vivo transformations that could alter their biological activities. These peptides are directly absorbable by the gastrointestinal tract, potentially supporting the protection and development of newborns.

The precursor proteins of endogenous peptides are crucial in determining their biological functions. Previous studies have identified CSN2, CSN1S1, and CSN3 as the primary sources of endogenous peptides, while proteins such as α-lactalbumin (α-LA), secretory immunoglobulin A (IgA), and lactoferrin (LF) were not associated with endogenous peptide production [23,50]. Our findings align with these studies, confirming CSN2 as the dominant precursor protein in buffalo milk across lactation stages. However, three novel endogenous peptides derived from lactoferrin were identified, suggesting that lactoferrin also contributes to endogenous peptide formation in buffalo milk. Furthermore, peptides derived from less-characterized precursor proteins, such as GLYCAM1 and M-SAA3.2, were identified. These proteins are notable for their roles in immune regulation. M-SAA3.2 is an acute-phase protein involved in modulating inflammatory responses and promoting tissue repair, while GLYCAM1 plays a role in cell adhesion and immune cell signaling. The presence of M-SAA3.2 and GLYCAM1-derived peptides in mature milk suggests these proteins may contribute to the immune-protective properties of buffalo milk. In contrast, buffalo colostrum does not rely on endogenous peptides from these proteins for immunomodulatory effects, emphasizing the distinct functional roles of peptides at different lactation stages.

Colostrum is widely recognized as a critical source of bioactive components that support neonatal immunity. This study provides new insights into the unique peptide profile of buffalo colostrum, which differs significantly from transitional and mature milk. Notably, 508 peptides exclusive to colostrum were identified, many of which originated from precursor proteins such as NEB, CIC, WIPF3, and FHOD3. These proteins are typically underrepresented in milk peptidomics, suggesting they have specialized roles in neonatal immunity. This finding is further corroborated by KEGG pathway analysis, which revealed significant enrichment of precursor proteins associated with immune-related pathways, including complement and coagulation cascades, NF-κB signaling, and Toll-like receptor signaling. These pathways are integral to innate immunity and provide rapid defense against microbial infections, highlighting the potential of buffalo colostrum as a functional food for immune support.

Buffalo colostrum is recognized as a significant source of bioactive peptides, with potential applications in pharmaceuticals and functional foods [5,51,52]. Compared to mature milk, buffalo colostrum is enriched with antibodies and bioactive peptides that protect newborns from pathogen infections, provide passive immunity, and support gastrointestinal and immune development [23,53]. The identification of 282 potentially bioactive peptides in buffalo colostrum represents a major advancement in the study of milk-derived bioactive peptides. However, translating these findings into practical applications requires further investigation. For instance, the 17 peptides predicted to possess anti-inflammatory properties could be isolated and tested in preclinical models of inflammatory diseases. Similarly, the 54 peptides with favorable water solubility could be incorporated into supplements to enhance their bioavailability.

Mammary epithelial cells produce substantial amounts of endogenous proteases, which are crucial in the generation of endogenous peptides [54]. Enzymatic cleavage analysis indicated that precursor proteins are likely cleaved by key enzymes, including cathepsin D, pepsin, trypsin, proline endopeptidase, elastase, plasmin, staphprotease, and others. Cathepsin D emerged as the most likely active enzyme in buffalo milk, with proline endopeptidase and plasmin functioning at specific lactation stages. Moreover, the characterization of the CSN2-derived endogenous peptide profile revealed that the identified peptides predominantly originated from four distinct regions: R_16_–S_33_, K_44_–E_106_, H_121_–Q_156_, and P_167_–V_224_. These enzymatic cleavage findings offer valuable insights into the mechanisms behind endogenous peptide production in buffalo milk, although experimental validation of the actual presence of these enzymes in buffalo milk is needed.

In bovine milk, CSN2, CSN1S1, and CSN3 are acidic phosphoproteins that undergo phosphorylation at Ser, Thr, and Tyr residues. Previous studies have documented phosphorylation sites in CSN2, CSN1S1, and CSN3 [23,55,56]. The number of phosphopeptides identified in this study surpasses that of earlier reports [50,57,58]. Phosphorylation of caseins facilitates the formation of organophosphate salts, which are essential for mineral delivery, particularly calcium [59]. In the present study, phosphorylation sites in CSN2 include S^30^, S^32^, S^33^, S^34^, S^37^, S^50^, T^135^, S^157^, T^169^, S^181^, S^183^, Y^195^, and Y^208^. Notably, S^34^, S^84^, S^111^, S^137^, and S^157^ are unique to mature milk. Similarly, phosphorylation sites in CSN1S2 include S^23^, S^24^, S^25^, S^28^, S^31^, T^128^, S^135^, T^136^, S^137^, S^141^, T^144^, and S^149^, with S^23^, S^25^, S^31^, T^128^, and S^135^ absent in colostrum. Phosphorylation sites in CSN3 include T^89^, S^89^, and S^216^, which were not detected in colostrum. These findings underscore the prevalence and functional significance of phosphorylation in buffalo milk proteins.

Endogenous peptides derived from CSN1S2 exhibit two distinct phosphorylation motifs. The first motif comprises five serine residues (positions: 23, 24, 25, 28, and 31) within a SerP-XXX-Glu/SerP sequence, while the second motif includes two serine residues (positions: 135 and 137) within a SerP-Thr-SerP sequence. Additionally, endogenous peptides from CSN1S2 exhibit six serine residues (positions: 30, 32, 33, 34, 37, and 50) within a SerP-XXX-Gln/SerP motif. These motifs highlight potential phosphorylation sites and emphasize the role of serine phosphorylation in regulating the functionality of CSN2 and CSN1S2 in buffalo milk. The phosphorylation pattern observed varies significantly among colostrum, transitional milk, and mature milk, suggesting that phosphorylation plays a regulatory role related to functional properties of these proteins at different lactation stages.

## 5. Conclusions

To our knowledge, this study provides a comprehensive peptidomic profiling of endogenous peptides in buffalo milk, revealing distinct endogenous peptide profiles in colostrum, transitional milk, and mature milk. Phosphorylation patterns and enzymatic cleavage sites elucidate peptide generation mechanisms. Furthermore, 17 bioactive peptides were characterized in colostrum, including anti-inflammatory candidates, highlighting its nutritional and therapeutic potential. These findings provide deeper insight into the molecular foundation underlying the functional properties of buffalo milk. To further explore the application value and confirm the physiological functions of these endogenous peptides, our future research will focus on conducting in-depth functional validation of these 17 characteristic peptides derived from colostrum.

## Data Availability

The original contributions presented in the study are included in the article/Supplementary Materials. Further inquiries can be directed to the corresponding authors.

## Supplementary Materials

The following supporting information can be downloaded at: https://www.mdpi.com/article/10.3390/foods15101728/s1, Supplementary File S1: The FDR curve and scatterplot of precursor mass error; Table S1: Peptide identification of database and de novo; Table S2: Precursor protein list; Table S3: N- and C-terminal of endogenous peptides; Table S4: Differential expression endogenous peptides; Table S5: Gene Ontology annotation; Table S6: KEGG pathway enrichment; Table S7: Candidate bioactive peptide list.
