# Supplementary material for: Peptidomic Profiling Analysis of Endogenous Peptides in Buffalo Milk During Lactation Stages
Source: Foods. 2026 May 14;15(10):1728. doi: 10.3390/foods15101728 (PMC13206046; doi:10.3390/foods15101728)
Supplement: Supplementary file 1 [file foods-15-01728-s001.zip › Supplementary File S1 The FDR curve and scatterplot of precursor mass error.pdf]

1. Experiment control of endogenous peptides in colostrum

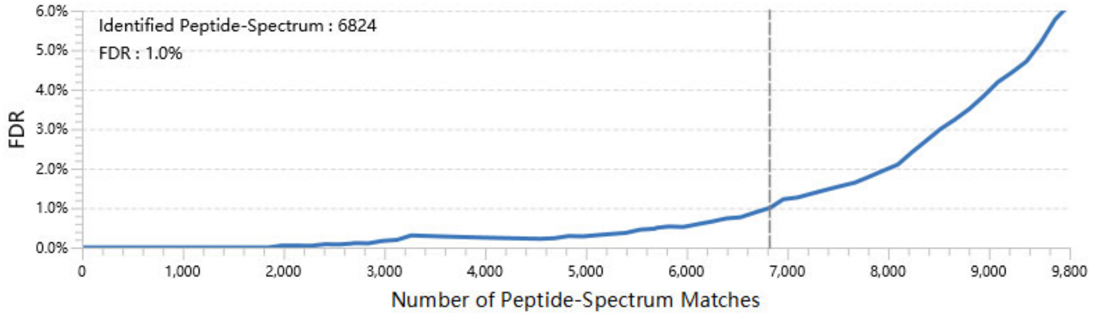

Figure S1-1. False discovery rate (FDR) curve. X axis is the number of peptides being kept. Y axis is the corresponding FDR.

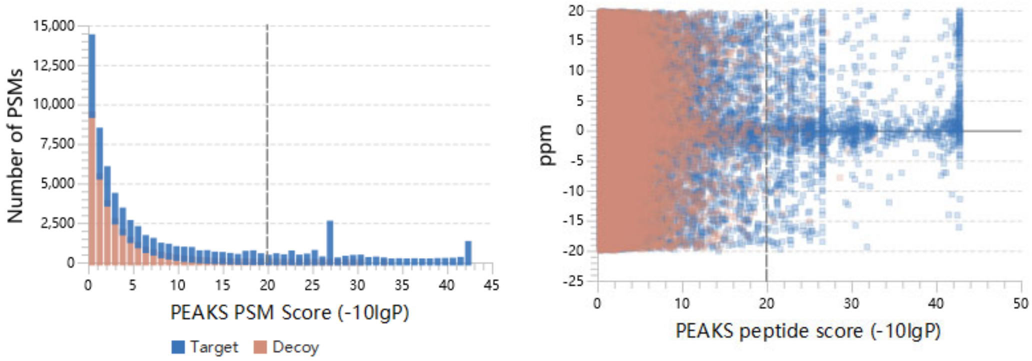

Figure S1-2. Score distribution.

(a) Distribution of PEAKS peptide score; **(b)** Scatterplot of PEAKS peptide score versus precursor mass error.

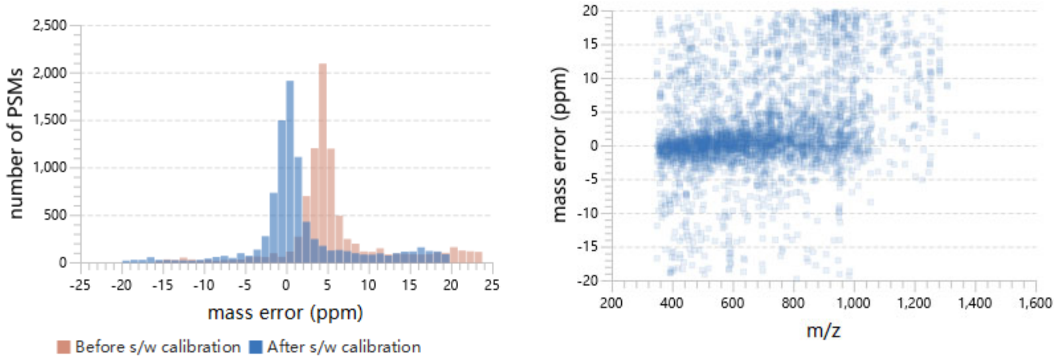

Figure S1-3. Precursor mass error of peptide-spectrum matches (PSM) in filtered result.

(a) Distribution of precursor mass error in ppm; **(b)** Scatterplot of precursor m/z versus precursor mass error in ppm.

2. Experiment control of endogenous peptides in transitional milk

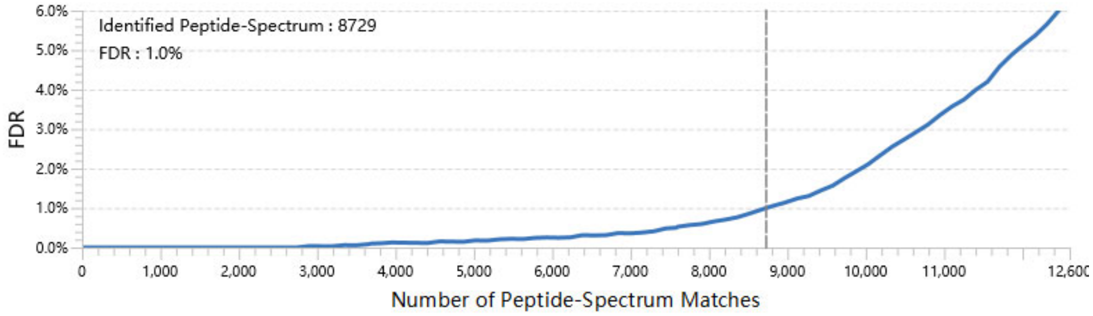

Figure S2-1. False discovery rate (FDR) curve. X axis is the number of peptides being kept. Y axis is the corresponding FDR.

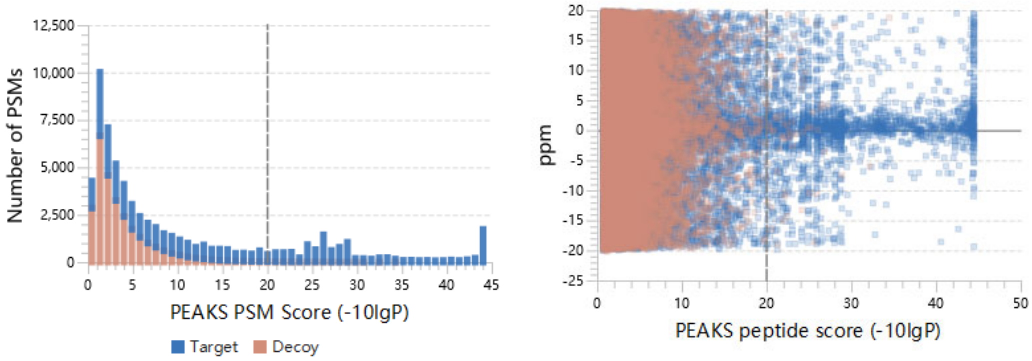

Figure S2-2. Score distribution.

(a) Distribution of PEAKS peptide score; **(b)** Scatterplot of PEAKS peptide score versus precursor mass error.

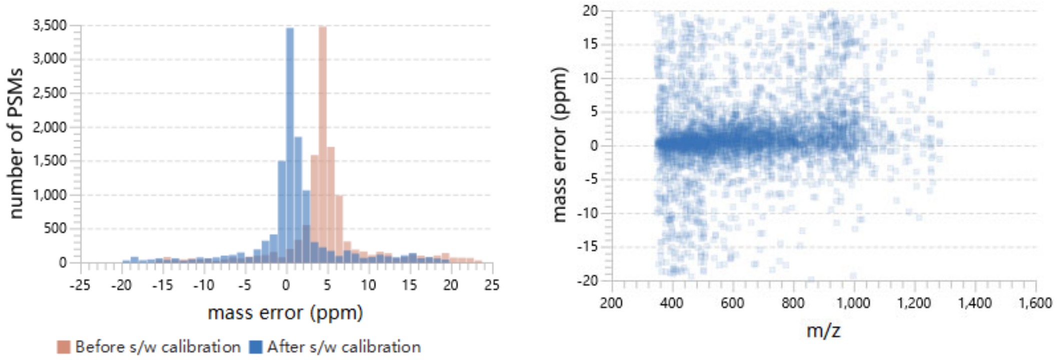

Figure S2-3. Precursor mass error of peptide-spectrum matches (PSM) in filtered result.

(a) Distribution of precursor mass error in ppm; **(b)** Scatterplot of precursor m/z versus precursor mass error in ppm.

3. Experiment control of endogenous peptides in mature milk

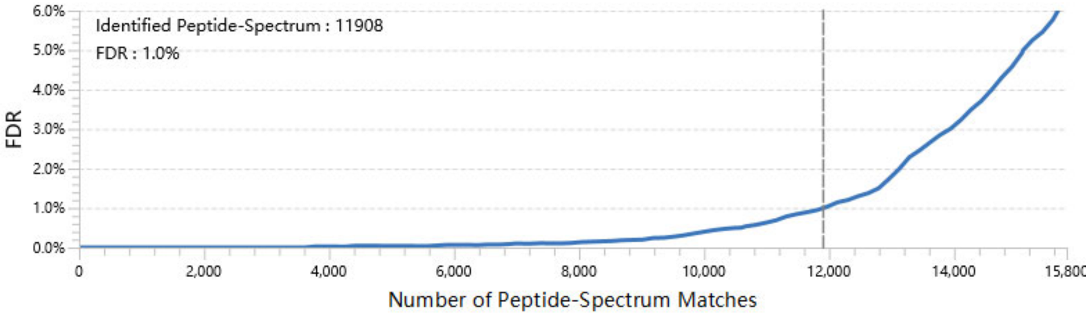

Figure S3-1. False discovery rate (FDR) curve. X axis is the number of peptides being kept. Y axis is the corresponding FDR.

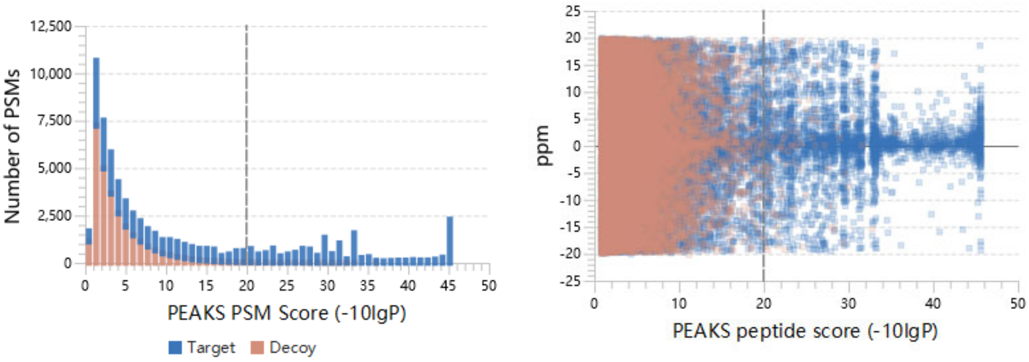

Figure S3-2. Score distribution.

(a) Distribution of PEAKS peptide score; **(b)** Scatterplot of PEAKS peptide score versus precursor mass error.

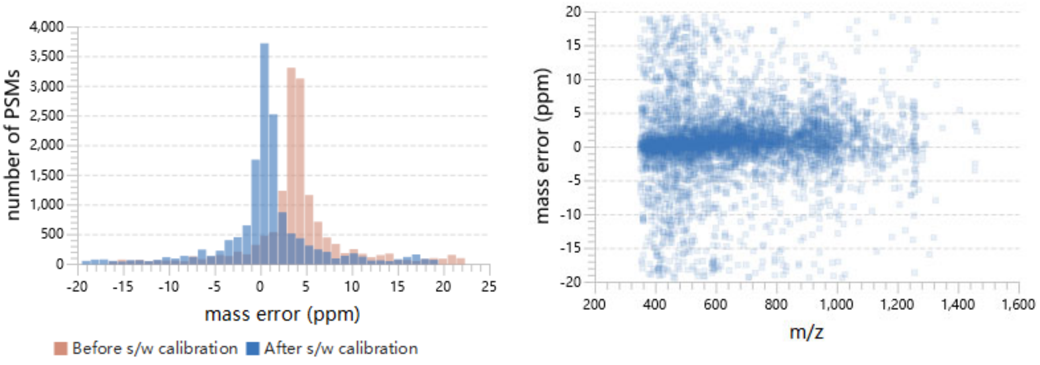

Figure S3-3. Precursor mass error of peptide-spectrum matches (PSM) in filtered result.

(a) Distribution of precursor mass error in ppm; **(b)** Scatterplot of precursor m/z versus precursor mass error in ppm.
